# Pericardial Decompression Syndrome and an Evanescent Tricuspid Regurgitation: A Real Conundrum

**DOI:** 10.7759/cureus.45571

**Published:** 2023-09-19

**Authors:** Mohammed-Bachir Mesfioui, Najlaa Belharty, Yousra Mesfioui, Nawal Doghmi, Mohamed Cherti

**Affiliations:** 1 Cardiology B Department, Ibn Sina University Hospital, Mohammed V University, Rabat, MAR

**Keywords:** echocardiography, acute tricuspid regurgitation, pericardial drainage, cardiac tamponade, pericardial decompression syndrome

## Abstract

Cardiac tamponade is a life-threatening condition that requires emergency pericardiocentesis. In rare cases, pericardial drainage may be followed by “pericardial decompression syndrome” (PDS), a poorly understood but potentially fatal syndrome characterized by acute ventricular dysfunction. It may present in different clinical forms of varying severity and be managed differently depending on the clinical context. In this article, we report an atypical presentation of this syndrome, with the development of laminar tricuspid regurgitation after pericardial drainage. To our knowledge, this complication has never been reported in the medical literature. Our understanding of the pathophysiology of this condition is based entirely on case reports. And because clinical studies are difficult to perform, the best defense against PDS is early detection so that it can be recognized and treated quickly.

## Introduction

Cardiac tamponade is a life-threatening condition that results from an increase in pericardial fluid, leading to cardiac compression and hemodynamic instability [1]. The treatment of cardiac tamponade involves drainage of the pericardial fluid, preferably by needle pericardiocentesis, with the use of echocardiographic or fluoroscopic guidance, and should be performed without delay in unstable patients to improve cardiac hemodynamics [2]. However, in approximately 5% of cases of percutaneously managed cardiac tamponade, patients may suffer “pericardial decompression syndrome” (PDS) [3], a poorly understood syndrome characterized by a paradoxical hemodynamic instability following pericardial drainage leading to ventricular dysfunction [4]. In this paper, we present an original presentation of this syndrome in a 53-year-old patient, admitted for a tamponade of tuberculous origin, whose drainage caused a massive tricuspid regurgitation that spontaneously resolved within a few days. To the best of our knowledge, this complication has never been reported in the medical literature. Our paper was written according to the CARE guidelines [5].

## Case presentation

**Figure 1:**
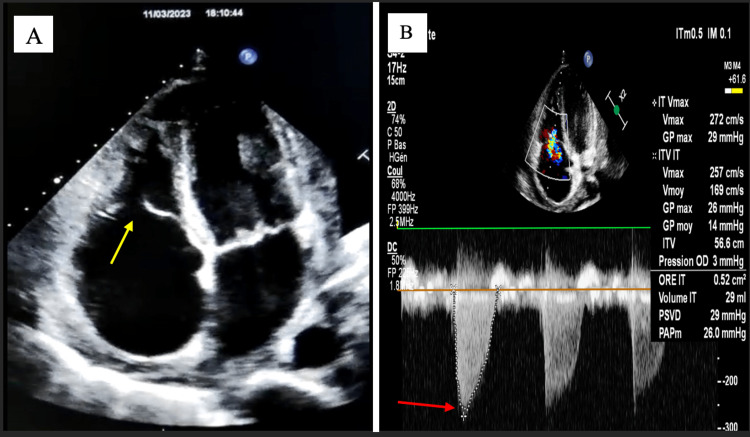
TTE findings one day after drainage A: Apical four-chamber view showing dilated right cavities with tricuspid valve diastasis (yellow arrow) B: Continuous wave doppler of the tricuspid regurgitation jet showing an envelope with triangular contour and early peak velocity (red arrow), which is indicative of severe tricuspid regurgitation

We report the observation of a 53-year-old patient, with a 30-pack-year history of smoking, who came to the emergency department for a two-week history of worsening dyspnea in a febrile context. His initial evaluation showed a blood pressure of 95/63 mmHg, sinus tachycardia at 160 bpm, and an oxygen saturation of 95% on room air. His lung fields were clear, jugular venous pressure was elevated, and the heart sounds were muffled. A chest X-ray demonstrated cardiomegaly, but otherwise, it was clear. An echocardiogram was performed, showing a left ventricle with preserved systolic function and a large circumferential pericardial effusion with tamponade physiology. An emergency needle pericardiocentesis was performed, and approximately 950 mL of serous fluid was removed. It was a lymphocytic exudate with elevated interferon-gamma (IFNG) levels, suggestive of tuberculosis. The tuberculous origin of the effusion was confirmed by GeneXpert testing, and treatment with anti-bacillary agents, aspirin, and colchicine was initiated. The patient was transferred to the intensive care unit with the drain in place. He initially did well, but the day after the procedure, he became hypotensive again and developed peripheral edema. He had no low output findings. The physical examination revealed an elevated jugular venous pressure with a prominent V wave and a mild hepatomegaly with a pulsatile liver. Another echocardiogram was performed, which showed an acutely dilated right ventricle with severe systolic dysfunction and dilatation of the tricuspid annulus, causing a massive tricuspid regurgitation (Figure 1; Video 1).

**Video 1:** TTE findings one day after drainage: apical four-chamber view showing laminar tricuspid regurgitation

**Figure 2:**
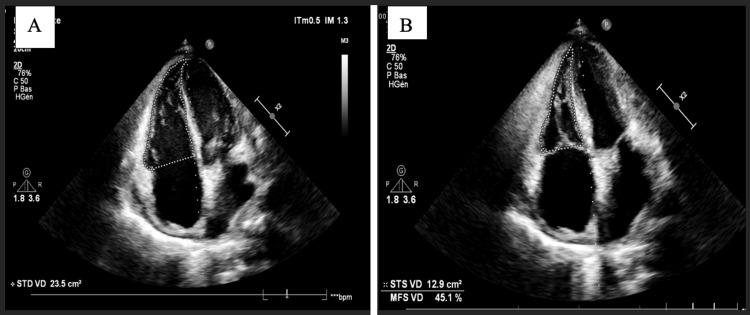
TTE findings three days later: apical view of the four cavities showing normal size and function of the RV with regression of regurgitation A: End-diastolic RV surface B: End-systolic RV surface RV, right ventricle

The clinical evolution was marked by the disappearance of the regurgitation in the following days with full recovery of the right ventricular function (Figure 2; Video 2). The patient was discharged home. A control echocardiography was performed one month later and showed no significant abnormality.

**Video 2:** TTE findings three days later: parasternal short-axis view showing resolution of the diastasis with normal coaptation of the tricuspid leaflets

## Discussion

PDS is defined as a paradoxical worsening of hemodynamics after uncomplicated pericardial drainage in patients with those with cardiac tamponade. It is also known as post-pericardial drainage low cardiac output syndrome, and it represents a rare complication with an incidence of about 5% but is severe with a described mortality of 16-29%. Mortality seems to be more important in the setting of surgical drainage [3,6]. The first description of this clinical syndrome dates back to 1983 [7] in a 45-year-old Caucasian patient with acute myeloid leukemia receiving surgical pericardiocentesis drainage of 500 mL of hematic fluid. However, it was not until 2010 that the term “pericardial decompression syndrome” was proposed [8]. In 2014, a literature review identified 35 cases, half following pericardiocentesis and half following pericardiotomy, with drained volumes ranging from 450 to 2100 mL. The clinical presentation is variable, ranging from acute pulmonary edema without shock to right, left, or bi-ventricular failure, and can occur up to 48 hours after the procedure [3]. The main risk factors for mortality are neoplastic involvement of the pericardium, post-radiation involvement, pericardial calcifications, prior impairment of myocardial function, or the need for circulatory support, whether drug or mechanical [9]. The pathophysiology is not fully understood at present. Several hypotheses have been put forward [10], and they are as follows:

- The hemodynamic hypothesis is based on an abrupt increase in venous return following the removal of hemodynamic obstruction from the effusion, resulting in increased afterload and right ventricular dilatation that may lead to right ventricular failure. The induced transmural pressure elevation of the right ventricle is further exacerbated by a negative intrapericardial pressure related to drainage. Therefore, the physiological pressure of the intrapericardial space being almost zero, the latter became negative following the opening of this space and the implementation of the drainage. The dilation of the right ventricle also leads to a deviation of the interventricular septum toward the left and, thus, a decrease in cardiac output by virtue of ventricular interdependence.

- The ischemic hypothesis suggests epicardial ischemia caused by decreased coronary flow in association with increased intrapericardial pressure during tamponade. The worsening of this ischemia after drainage is possibly explained by the increase in transmural pressure described above.

- The last hypothesis suggests a neuro-vegetative origin with an abrupt decrease in sympathetic activity after drainage, which may unmask a pre-existing ventricular dysfunction or lead to para-sympathetic hyperactivity.

In the absence of specific treatment, management is essentially based on early diagnosis and the introduction of hemodynamic support therapy. The introduction of a vasopressor or inotropic treatment is often necessary, and the most severe cases may justify the introduction of external circulatory assistance [11]. In the literature, the time it takes for a functional myocardial recovery is quite variable. While the two patients reported by Wolfe et al. required one week and two weeks each for restoration to their baseline cardiac function [12], the patient reported by Vandyke et al. was able to be weaned off of all inotropic and ventilator assistance four days following her decompensation [7]. In our intriguing situation, hypothetically, the combination of hemodynamic and ischemic theories would explain the right ventricular failure: the consequent massive tricuspid regurgitation and its resolution. Right ventricular enlargement due to increased venous return and right myocardial stunning due to the impairment of coronary blood flow resulted in annular dilatation and tricuspid regurgitation. The spontaneous resolution of the leak in a few days testifies to its purely functional character despite its laminar nature.

## Conclusions

PDS is rare but serious with a high mortality rate. Close hemodynamic monitoring is strongly advised during the first day post-drainage, and transthoracic echocardiography should be performed immediately if clinical deterioration occurs. However, this syndrome can have some particular presentations, like the one highlighted in our observation. Given the paucity of data on the subject, management is decided on a case-by-case basis and depends on the clinical presentation; it ranges from close monitoring in cases of good hemodynamic tolerance to hemodynamic support therapies with circulatory assistance in extreme cases.
